# Comparative Analyses of Muscle Quality in Hooked, Trawl-Net, and Radar-Net Hairtail (*Trichiurus haumela*) during Thermal Processing

**DOI:** 10.3390/foods13183005

**Published:** 2024-09-22

**Authors:** Wenxiong Zheng, Ronglin Yang, Shanshan Shui, Hongbo Yan, Jia Song, Xiaoguo Ying, Soottawat Benjakul, Bin Zhang

**Affiliations:** 1 College of Food Science and Pharmacy, Zhejiang Ocean University, Zhoushan 316022, China; 13242662201@163.com (W.Z.); 18924444970@163.com (R.Y.); 19818085800@163.com (H.Y.); 19883835696@163.com (J.S.); yingxiaoguo@zjou.edu.cn (X.Y.); zhangbin@zjou.edu.cn (B.Z.); 2 Department of Food Science and Nutrition, College of Biosystems Engineering and Food Science, Zhejiang University, Hangzhou 310058, China; 3 Pisa Marine Graduate School, Zhejiang Ocean University, Zhoushan 316022, China; 4 International Center of Excellence in Seafood Science and Innovation, Faculty of Agro-Industry, Prince of Songkla University, Songkhla 90110, Thailand; soottawat.b@psu.ac.th

**Keywords:** hairtail, thermal processing, muscle quality, microstructure

## Abstract

To investigate and compare the changes in muscle quality of hooked, trawl-net, and radar-net hairtail (*Trichiurus haumela*, HH, TH, and RH) during thermal processing, the physicochemical properties of three kinds of hairtail were determined under heating at 30, 50, 70 and 90 °C for 10 min. Additionally, the muscle tissues were observed via Oil Red O (ORO) staining, Masson staining, and scanning electron microscopy (SEM). The results showed that with increased heating temperature, pH, L*, b*, chewiness, and gumminess in hairtail muscle increased, while a* and shearing force decreased. The springiness, relative contents of hydrophobic and disulfide bonds, myosin surface hydrophobicity, and TCA-soluble peptide content increased first and then decreased. However, the relative contents of ionic and hydrogen bonds showed an opposite trend. Histological observations revealed that heating disrupted hairtail muscle tissue, manifested by the blurriness and disorder of myofibrils and breakage of myofibrillar bundle membranes. The RH muscle exhibited the highest chewiness, gumminess, and chemical force levels, accompanied by the lowest content of TCA-soluble peptide. Furthermore, the RH muscle presented the greatest fat droplet content, diffusivity, and integrity of collagen and myofibers. Correlation analysis revealed a close correlation between muscle quality and protein function in HH, TH, and RH. This study provides a theoretical basis for the difference in muscle quality in three different types of hairtail.

## 1. Introduction

Hairtail (*Trichiurus haumela*), as a commercial marine fish, is widely distributed in the eastern Pacific and the Indian Ocean [1]. Hairtail is favored by consumers because of its rich nutritional contents of protein and lipids, especially polyunsaturated fatty acids, e.g., terephthalic acid (TPA), docosahexaenoic acid (DHA), and eicosapentaenoic acid (EPA) [2].

Based on the fishing method, hooked hairtail (HH), trawl-net hairtail (TH), and radar-net hairtail (RH) are the three main hairtails in Zhejiang Province, China, which all belong to the genus Japanese hairtail (*T. haumela*). HH is caught using specialized hooks and lives closer to the shore in the shallow ocean. RH is caught by radar nets and lives in ocean depths of 50–100 m. Unlike the above two types of hairtail caught in Zhoushan’s inner ocean, TH is caught in the North Ocean with a trawl net. The first capture method is gentler than the latter two but needs to be more efficient and yields less. The appearance integrity of RH is the lowest among the three kinds of hairtail due to its violent struggle during fishing. However, the deep-sea living area results in the rich lipid content and compact muscle of RH [3].

The catching methods and living area affected the muscle quality of aquatic products. Huo et al. reported that the neon flying squid (*Ommastrephes bartramii*), jumbo squid (*Dosidicus gigas*), and Argentine shortfin squid (*Illex argentinus*), living in different sea areas, exhibited significantly different physicochemical and volatile flavor properties during chilled storage [4]. Aside from the living area, there are also several previous studies on the influence of the fishing method on the muscle quality of the aquatic product. For example, Sogn-Grundvag et al. verified that the fishing method impacted the quality of Atlantic cod and haddock [5]. Similarly, Raoofi et al. revealed that the beach seine method had fewer adverse effects on the muscle quality of *Rutilus kutum* compared to gillnet fishing [6]. However, in addition to catch methods, the flavor, texture, and nutrition of hairtail products are also prone to deterioration depending on the processing conditions, e.g., programmed temperature, environmental pH, and solvent concentration [7]. In particular, processing methods significantly impact the quality of fish and its products [8].

Heat processing is one of the universal technologies for processing fish, shrimp, and their products, which can impact the nutrition, quality, and safety of fish muscle by changing the texture, color, flavor, lipid, and protein properties [9]. Singh and Benjakul indicated that the endogenous heat-activated proteases in fish or shellfish were activated during thermal processing. Then, the degradation of the myofibrillar proteins was catalyzed, weakening the water-holding capacity and the muscle gel [10]. Sreenath et al., found that the increased exposure time of thermal treatment caused a decrease in lightness (L*), yellowness (b*), and shear value, and an increase in redness (a*) in the muscle of Indian white shrimp (*Fenneropenaeus indicus*), which subsequently affected the shrimp muscle quality [11]. Ahtisham et al. detected that 90 °C heat treatment prior to canning reduced water-holding capacity, water content, heating yield, and the toughness of the texture in Atlantic mackerel (*Scomber scombrus*) [12]. Jiang et al., found that thermal processing resulted in excessive cooking losses and the denaturation of myofibrillar and sarcoplasmic proteins in the big-head carp (*Aristichthys nobilis*) [13]. Jiang et al. showed that the cooking loss increased significantly with increasing temperature and heating time, the content of myosin heavy chains decreased to disappear completely, and the actin content reduced significantly. Furthermore, the content of water-soluble proteins increased significantly, indicating that cooking at high temperatures for a long time resulted in the degradation of muscle proteins, leading to a worsened texture and quality deterioration [14]. At present, thermal processing technology is widely used in aquatic products because of its convenience and high sterilization efficiency. However, the thermal processing of hairtail has not been reported, especially in comparative studies of muscle quality in different kinds of hairtail.

In our previous research, HH, TH, and RH were water-bathed at 30, 50, 70, and 90 °C for 10 min. The cooking loss, water-holding capacity (WHC), myosin turbidity, solubility, emulsifying capacity, foaming capacity, foam stability, and carbonyl content were determined. The results verified that the muscle quality, including myosin functional properties of all three kinds of hairtail, decreased during thermal processing, and the muscle properties of RH were the most stable.

The current study aimed to investigate the physicochemical properties (pH, color, textural properties, chemical force contents, myosin surface hydrophobicity, and TCA-soluble peptide content) in HH, TH, and RH muscles during thermal processing. Moreover, histological changes in muscles in three types of hairtail at 50 °C and 90 °C were determined by Oil Red O (ORO) staining, Masson staining, and SEM. The results will provide basic knowledge and references for applying heat processing technology in different kinds of hairtail.

## 2. Materials and Methods

### 2.1. Chemical Reagents

Sodium chloride (NaCl), sodium hydroxide (NaOH), trichloroacetic acid (C_2_HCl_3_O_2_), bromophenol blue, urea, Tris-HCl buffer, ethylene glycol bis (C_2_H_6_O_2_) and β-mercaptoethanol were purchased from fiSinopharm Chemical Reagent Co., Ltd. (Suzhou, China). All reagents were of analytical grade.

### 2.2. Hairtail Samples and Treatments

Fresh HH, TH, and RH (width of 4 ± 0.5 cm, length of 0.7 ± 0.1 m, and weight of 500 ± 50 g) were purchased from Zhoushan International Fisheries Market (Zhoushan, China). The hairtail samples were placed in a foam box filled with crushed ice and transported to the laboratory within half an hour. Upon arriving at the laboratory, the hairtails were washed with ice water. Additionally, the head and viscera of the hairtail samples were manually removed. The skin and the red and white muscles of three kinds of hairtail were kept. Subsequently, the hairtails (close to the middle place) were divided into 5–6 cm pieces (width of 4 ± 0.5 cm, length of 5.5 ± 0.5 cm, and weight of 70 ± 10 g), placed in sealed pockets, and subjected to heating at 30, 50, 70, and 90 °C for 10 min in a water bath. Following this, hairtail samples were brought to normal temperature, and the physicochemical characteristics of three types of hairtail from the same part were measured in triplicate. However, the hairtail samples were not used up on the same day. The remaining hairtail samples were cryopreserved at −80 °C and pre-thawed at 4 °C for 3 h before experiments.

### 2.3. Measurement of pH

The pH determination measure was created by Wang et al. [15]. Hairtail samples (5.00 g) were stirred with 45 mL deionized water and rested for 1 h, followed by collection of the supernatant. A PHS-3C pH meter (Changzhou Depo Textile Technology Co., Ltd., Changzhou, China) was used to determine the pH of the supernatant.

### 2.4. Determination of Color

The color of the hairtail muscle was determined based on the approach of Jiao et al. [16]. A CR-10 colorimeter (Konica Minolta Investment Ltd., Shanghai, China) was used to determine the L*, a*, and b* values of hairtail muscle. Notably, a positive value of L* indicated whiteish, while a negative value of L* indicated blackish; a positive value of a* indicated reddish, while a negative value of a* indicated greenish; and a positive value of b* indicated yellowish, while a negative value of b* indicated blueish colors.

### 2.5. Textural Properties Analyses

The textural properties were determined in accordance with the methodology proposed by Yang et al. [17]. Fish maws of hairtail samples were placed on a physical property food analyzer (TMS-PRO, FTC, Sterling, VA, USA). The Texture Expert Exceed version 1.22 computer program by Stable Micro System was used to collect data and perform calculations. From the TPA curves, the texture parameters including springiness, chewiness, gumminess, and shearing force were obtained. Textural property parameters were determined in the following conditions: P36/R cylindrical Perspex probe (4 mm in diameter), constant test speed of 60 mm/min, backhaul rate of 60 mm/min, backhaul height of 25 mm, and a compression ratio of 50%. The above experimental operation was repeated six times to gain six parallel data sets.

### 2.6. Chemical Force Content Analyses

The relative contents of ionic bond, hydrogen bond, hydrophobic interaction, and disulfide bond chemical forces were determined following the method by Deng et al. [18]. The chemical bond content was quantified as the relative mass fraction of proteins dissolved in the buffer, following the protein determination approach from research by Lowry et al. [19].

### 2.7. Myosin Extraction and Surface Hydrophobicity Analysis

The extraction of myosin was conducted in accordance with the methodology outlined by Gao et al. [20]. The myosin concentration (0.1 mg/mL) was determined using the biuret method, and samples were stored at 4 °C for future analyses.

The surface hydrophobicity was determined using the approach by Toru et al. [21]. A solution of myosin at a concentration of 0.1 mg/mL (2 mL) was combined with 20 μL of 32 mM 8-aniline-1-naphthalene sulfonate (ANS, a fluorescence probe) and incubated at room temperature for 10 min to allow the formation of ANS–protein complexes. The fluorescence intensity was quantified using a spectrofluorophotometer (RF-5300PC, Shimadzu, Kyoto, Japan).

### 2.8. Determination of TCA-Soluble Peptide Content

The TCA-soluble peptide content was measured, referring to the approach of Zhu et al. [22]. Hairtail samples (3 g) were homogenized with 27 mL of 5% TCA (*w/v*) at 12,000× *g* (Tube Control 100, IKA, Staufen, Germany). After standing at 4 °C for one hour and stirring occasionally, the mixture was centrifuged at 12,000× *g* for five minutes. The TCA-soluble peptide content was measured according to the method by Lowry et al. [19].

### 2.9. Histological Change Analysis

Oil Red O (ORO) staining, Masson staining, and scanning electron microscopy (SEM) analyses were used to evaluate the microstructural changes in the muscle tissues of three kinds of hairtail during thermal processing. Samples were stained by Oil Red O and hematoxylin as described in research by Saravolac et al. [23], and then analyzed and photographed via light microscopy (BX51, Olympus, Japan) as described in a previous study by Shui et al. [24]. The Masson staining was performed according to the report by Fan et al. [25]. SEM was performed referring to the procedure of Nawaz et al. [26]. Samples stained with ORO and Masson were observed at a 200× magnification, while SEM samples were observed at a 4000× magnification.

### 2.10. Data Analysis

Data were presented as the means ± standard deviation (SD) of the measurements for 3 replicates (n = 3), except for the determination of textural properties (six parallel measurements). The SPSS v26.0 software package (SPSS Inc., Chicago, IL, USA) was used for statistical analyses. Then, the significance at the level of *p* < 0.05 was determined by Duncan’s test. Pearson correlation analysis between muscle quality and protein function was conducted utilizing Origin 2022 (Origin-Lab Corp., Northampton, MA, USA).

## 3. Results and Discussion

### 3.1. pH Value

**Figure 1:**
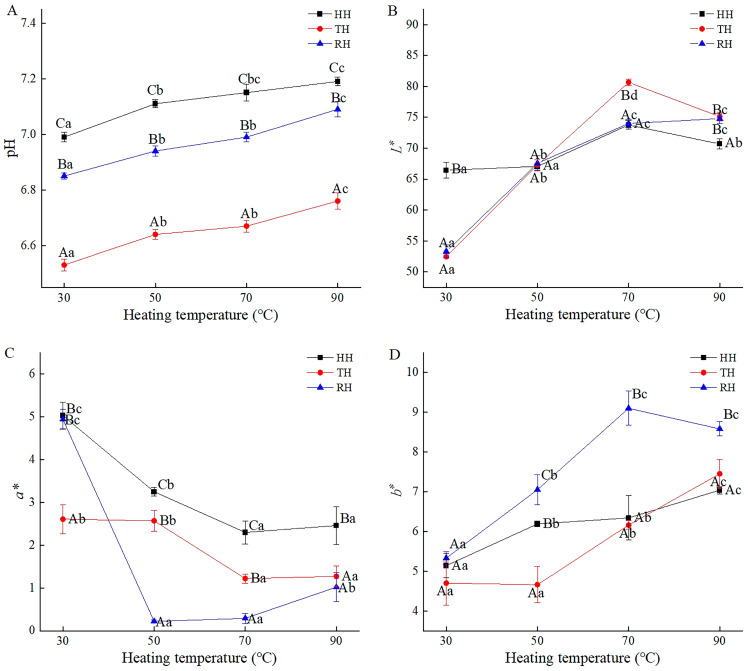
pH value (**A**), L* (**B**), a* (**C**), and b* (**D**) in hairtail muscles during thermal processing. HH, hooked hairtail; TH, trawl-net hairtail; RH, radar-net hairtail. Different lowercase letters in different heating temperatures for the same hairtail sample indicate significant differences (*p* < 0.05), and different uppercase letters in different hairtail samples for the same heating temperature indicate significant differences (*p* < 0.05).

pH is a critical index for evaluating aquatic muscle freshness. The limit of acceptability in post-mortem pH is usually 6.8–7.0 [27]. According to previous research, the pH in the fresh muscle of HH, TH, and RH was 6.89, 6.72, and 6.82 (*p* > 0.05), respectively [3]. As shown in Figure 1A, with an increase in heating temperature from 30 °C to 90 °C, the pH value in three kinds of hairtail showed an upward trend. The high-temperature process destroyed the protein stability by changing the protein tertiary structure, inducing a reduction in the content of acidic groups and an increase in pH [28]. Increased pH was also likely due to the nitrogenous compounds decomposing and alkaline compounds accumulating, such as ammonia [29]. Ah-Na et al. speculated that the increase in pH mainly resulted from the cleavage of bonds in the imidazole, sulfhydryl, and hydroxyl groups of various amino acids in muscle protein and the release of free hydroxide ions [30]. At the same heating temperatures of 30, 50, 70, and 90 °C, the value of pH in TH muscle was significantly lower than that of HH and RH muscles (*p* < 0.05), which was likely attributable to the low content or activity of endogenous enzymes and little production of alkaline compounds in TH [31].

### 3.2. Color Characteristics

The color difference is critical in evaluating the appearance and deterioration degree of aquatic products. Generally, aquatic products with relatively high L* and a* values are more accepted by customers. The results of previous research showed that the L*, a*, and b* values of all the fresh hairtail muscles were in the range of 59.36–69.66, 1.95–2.59, and 6.19–7.79, respectively, and they exhibited a bright red color [3]. As presented in Figure 1B–D, the L* and b* of three kinds of hairtail muscles showed an overall uptrend, and the a* tended to decrease. The increased L* and b* indicated that the hairtail muscle turned bright and yellow, while the decrease in a* suggested that the hairtail muscle became green. These results partly conformed to research by Monika et al., on pikeperch fillets [32]. Heating treatment led to the denaturation and coagulation of myosin and the damage of pigment proteins, such as ferroheme [33], associated with increased L* and b*. Myosin turbidity in three kinds of hairtail increased during the heating process in our previous study, demonstrating myosin degeneration [34]. At the same time, ferrous myoglobin was oxidized to high-iron myoglobin in the air when heated in a water bath, decreasing the a* value [35]. Above 70 °C, the change in L*, a*, and b* was more steady in the HH and RH samples, likely because the content of myosin and pigmented protein was greatly reduced, and the protein denaturation reaction gradually stopped [36]. Notably, RH showed the lowest a* value and the highest b* value at 90 °C, indicating that the color of RH muscle turned green and yellow, which was likely associated with the content and arrangement of protein in RH muscle.

### 3.3. Texture Property

**Figure 2:**
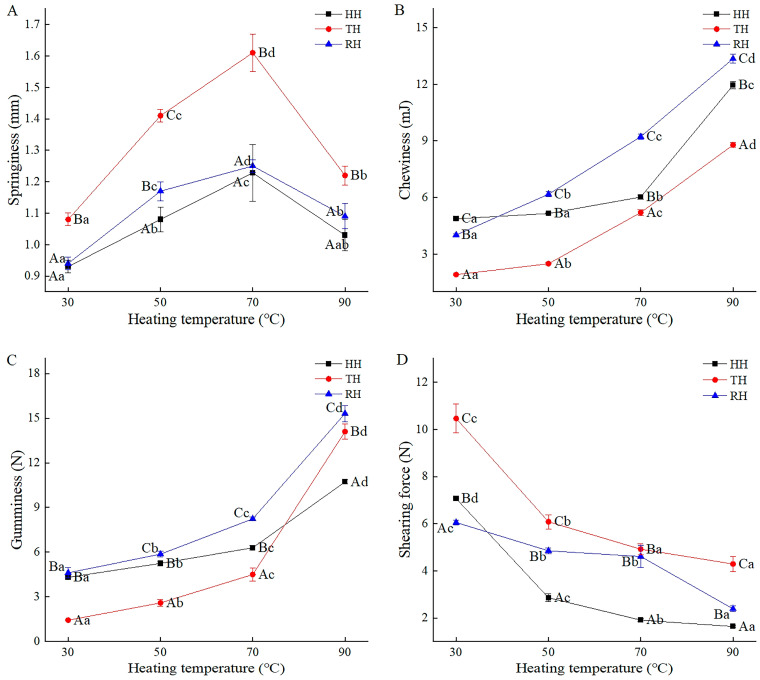
Springiness (**A**), chewiness (**B**), gumminess (**C**), and shearing force (**D**) in hairtail muscles during thermal processing. HH, hooked hairtail; TH, trawl-net hairtail; RH, radar-net hairtail. Different lowercase letters in different heating temperatures for the same hairtail sample indicate significant differences (*p* < 0.05), and different uppercase letters in different hairtail samples for the same heating temperature indicate significant differences (*p* < 0.05).

Texture can directly express the tissue state, structural change, and sensory quality of fish, which was related to the myofibrillar proteins and collagenous deforming [37]. According to previous research, the springiness, chewiness, gumminess, and shearing force values of all the fresh hairtail samples were in the range of 0.91–0.98 mm, 4.95–7.60 mJ, 5.24–7.96 N, and 6.92–8.83 N, respectively. In the current study, the changes in springiness, chewiness, gumminess, and shearing force in three kinds of hairtail under heating of 30, 50, 70, and 90 °C are shown in Figure 2. During the heating process, the springiness in the three kinds of hairtail tended to rise first and then decline. The chewiness and gumminess increased, while the shearing force decreased. The increase in springiness was associated with the loss of water and the thermal coagulation of protein [38]. Then, the heat promoted the destruction of the spatial structure of myosin and the dissolution of soluble protein compounds above 70 °C, leading to the loosening of the myofibrillar network and the decrease in springiness in HH, TH, and RH. The results were partially consistent with the research on sturgeon fillets by Cai et al. [37]. Furthermore, the myofibrillar protein denatured and the collagen protein contracted at high temperatures, causing the enhancement of cell binding force and a close bind of myofibrillar protein, which increased chewiness and gumminess and decreased shearing force [37,39]. Similar observations were found in abalone muscles with heat treatment [40]. The springiness and shearing force in the TH muscle were higher than those in HH and RH, possibly due to the higher elastin content in the TH sample. Meanwhile, because of the more stable muscle structure, the closer myofibril arrangement, and the greater water-holding capacity in the RH, the chewiness and gumminess in the RH sample were the highest, and the shearing force change was the smallest. Analogous texture changes could be found in the study of half-shell scallops by Zhan et al. [41].

### 3.4. Chemical Force Content

**Figure 3:**
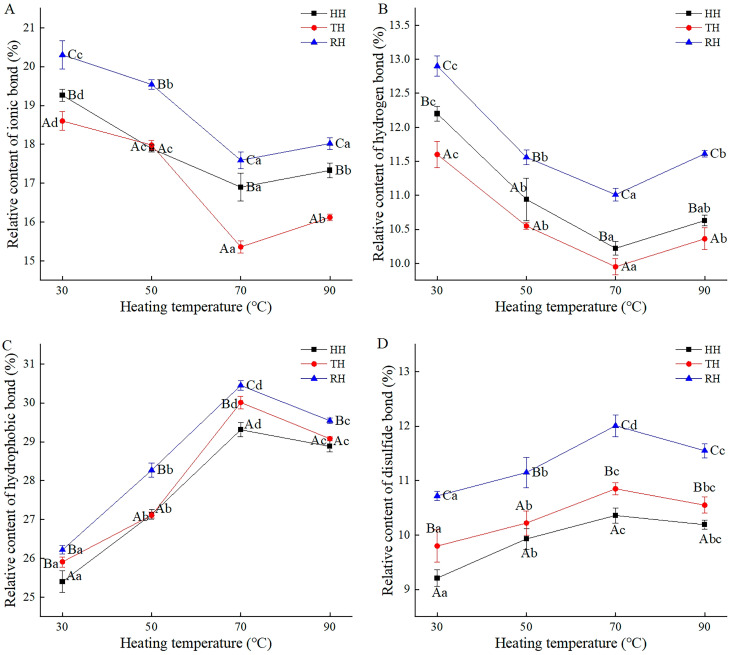
Relative contents of ionic bonds (**A**), hydrogen bonds (**B**), hydrophobic bonds (**C**), and disulfide bonds (**D**) in hairtail muscles during thermal processing. HH, hooked hairtail; TH, trawl-net hairtail; RH, radar-net hairtail. Different lowercase letters in different heating temperatures for the same hairtail sample indicate significant differences (*p* < 0.05), and different uppercase letters in different hairtail samples for the same heating temperature indicate significant differences (*p* < 0.05).

Chemical force content reflects the stability degree of protein conformation and the gel characteristics in the fillet. The change in chemical force contents in hairtail myosin implied a change in the structure and molecular size of proteins, which possibly promote the digestion and absorption of protein in the human body. In our previous study, the relative content of hydrogen bonds, ionic bonds, hydrophobic bonds, and disulfide bonds in the fresh muscle of three kinds of hairtail were measured in the range of 46.46–51.80%, 34.12–37.51%, 8.77–14.87%, and 8.64–20.56%, respectively. As shown in Figure 3, the content of ionic bonds and hydrogen bonds in HH, TH, and RH samples decreased below 70 °C and then increased. However, there was an opposite tendency in the relative content of hydrophobic bonds and disulfide bonds. The relative content of chemical bonds was connected to the change in protein conformation after thermal treatment [42]. The change in chemical force contents also led to the destruction of the myosin functional properties. Kunarayakul et al. reported that the decrease in the content and formation ability of hydrogen bonds between amino and carboxyl groups of protein molecules led to the weakness of the foaming capacity and foaming stability of the myosin during thermal processing [43]. Our previous research confirmed that the foaming capacity and foaming stability of myosin continuously presented a downward trend below 70 °C, which was partly consistent with this conclusion. Above 70 °C, the degradation of protein and the production of new substances, such as water, carbon dioxide, and amines, brought about the formation of ionic bonds and hydrogen bonds [44]. Meanwhile, the heat treatment brought the rupture and breakdown of the hydrophobic bonds and disulfide bonds in protein [45]. Furthermore, the relative contents of all chemical bonds in RH muscle were significantly higher than those in HH and TH heated at the same temperature (*p* < 0.05). These might be due to the tight and orderly arrangement of the muscle fibers in the RH sample.

### 3.5. Surface Hydrophobicity

**Figure 4:**
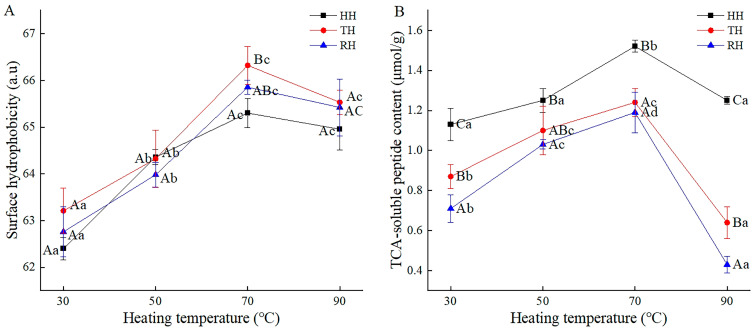
Myosin surface hydrophobicity (**A**) and TCA-soluble peptide content (**B**) in hairtail muscles during thermal processing. HH, hooked hairtail; TH, trawl-net hairtail; RH, radar-net hairtail. Different lowercase letters in different heating temperatures for the same hairtail sample indicate significant differences (*p* < 0.05), and different uppercase letters in different hairtail samples for the same heating temperature indicate significant differences (*p* < 0.05).

Surface hydrophobicity is commonly used to assess protein surface activity and characterize protein conformation changes. As determined in a previous study, the surface hydrophobicity of myosin in the fresh muscle of HH, TH, and RH was 50.55, 50.4, and 48.7 a.u. The current results shown in Figure 4A indicate that the myosin surface hydrophobicity of HH, TH, and RH samples increased and reached a maximum of 65.30 a.u, 66.32 a.u, and 65.85 a.u at 70 °C, respectively. The heat-induced denaturation of myosin, expansion of the peptide chain, and exposure of hydrophobic groups might upregulate surface hydrophobicity, as reported by Toru et al. [21]. Jiang et al., also found that the myosin denatured in *Aristichthys nobilis* after heating treatment according to SDS-PAGE analysis [14]. Additionally, the increase in myosin turbidity and the decrease in myosin solubility in our previous research verified the denaturation of myosin [34]. Furthermore, surface hydrophobicity tended to be stable above 70 °C, which may be attributed to the polymerization of hydrophobic groups and the encapsulation of some hydrophobic amino acid residues [46]. The results are consistent with the decreased trend in myosin EAI and ESI in hairtail muscle of the previous results. Moreover, the surface hydrophobicity of TH in the current study and the EAI and ESI of TH in the previous study were slightly higher than those of HH and RH. According to the research by Mohan et al. and Encinas-arzate et al. [47,48], it was speculated that the hydrophobic groups in muscle proteins of HH and RH were damaged more seriously, which led to weakness in the crosslinking ability of protein and fat particles and a decrease in protein functional characteristics, such as EAI and ESI [34].

### 3.6. TCA-Soluble Peptide Content

TCA-soluble peptide content expresses the protein hydrolysis degree. Previous research showed that the TCA-soluble peptide content in fresh samples of HH, TH, and RH was 1.13, 0.87, and 0.71 μmol/g. As presented in Figure 4B, the TCA-soluble peptide content in HH, TH, and RH muscles increased by 0.39, 0.37, and 0.48 μmol/g from 30 °C to 70 °C and then decreased by 0.27, 0.6, and 0.76 μmol/g at 90 °C, respectively. During heating, heat-resistant enzymes in the range of 50–60 °C in the muscle aggravated protein hydrolysis, increasing the content of TCA-soluble peptides [49]. The decrease in myosin solubility in the previous study also implied a reduction in myosin, which partially verified the explanation above. Above 70 °C, the thermal decomposition of soluble peptides led to the destruction of peptide chains and the production of amino acids, with the TCA-soluble peptide content decreasing [50]. A high chemical bond content is generally required for a stable protein structure and a low TCA-soluble peptide content [51]. At the same temperature, the lowest TCA-soluble peptide content in the RH sample was consistent with the above chemical bond results. The lower degree of protein denaturation was attributed to the more stable protein structure in RH muscle.

### 3.7. Histological Changes

**Figure 5:**
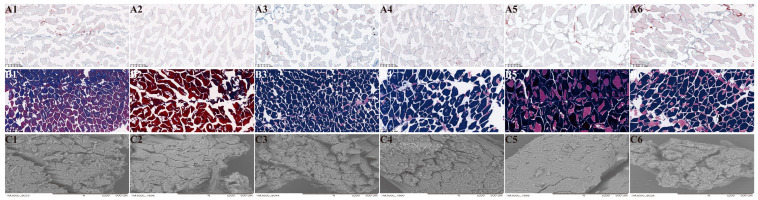
Histological microstructures of hairtail muscle tissues. (**A1**–**A6**): ORO staining micrographs; (**B1**–**B6**): Masson staining micrographs; and (**C1**–**C6**): scanning electron microscopy (SEM) micrographs. (**A1**,**B1**,**C1**): hooked hairtail (HH) heated at 50 °C for 10 min; (**A2**,**B2**,**C2**): trawl-net hairtail (TH) heated at 50 °C for 10 min; (**A3**,**B3**,**C3**): radar-net hairtail (RH) heated at 50 °C for 10 min; (**A4**,**B4**,**C4**): HH heated at 90 °C for 10 min; (**A5**,**B5**,**C5**): TH heated at 90 °C for 10 min; (**A3**,**B3**,**C3**): RH heated at 90 °C for 10 min.

Observations of the microstructure of hairtail muscle tissues are presented in Figure 5, including ORO staining, Masson staining, and SEM analysis. As shown by the ORO staining results (Figure 5(A1–A6)), cell nuclei (blue staining) and lipid droplets (red staining) in the gaps in the three kinds of hairtail muscles were not obviously observed. This result was partially consistent with the report by Shui et al. [24]. In addition, the distribution, quantity, and morphology of lipid droplets were not significantly different among all samples. Though lipid droplets in the RH sample were not clear at 50 °C, a larger diffusion occurred at 90 °C, potentially attributed to the increase in molecular gaps under heat stress.

Furthermore, the blue staining (collagen) and red staining (myofibrils) in the three kinds of hairtail muscle microstructure were further observed under a microscope via Masson staining (Figure 5(B1–B6)) [24]. Compared to a temperature of 90 °C, more tightly connected myofibrils and collagen were found in the muscle tissues of TH, HH, and RH at 50 °C. When the temperature rose to 90 °C, some of the myofibrils in the RH were more steady and orderly with larger collagen droplets compared with TH and HH. The space (white area) between myofibrils in HH muscle was significantly enlarged, and the collagen content was lower than that of TH and RH. A similar result and conclusion could be found in HE staining in the previous study, which indicated that the muscle fiber gaps of the three kinds of hairtail were the largest at 90 °C.

The SEM microstructures of muscle tissue in HH, TH, and RH were obtained at 50 °C and 90 °C. With the increase in heating temperature, the myofibrils in three kinds of hairtail tissues were expanded, leading to large lacunae and protein denaturation. Similar results and explanations can be found in the research by Cai et al. [37]. The membrane between the bundles of muscle fibers in all samples was entirely disrupted, and the phenomenon of granulation gradually formed on the surface of the membrane at 50 °C. Rupturing of the membranes and endomembranes of muscle fiber bundles occurred with intensified denaturation of sarcoplasmic proteins at 90 °C.

### 3.8. Correlation Analysis between Muscle Quality and Protein Function

**Figure 6:**
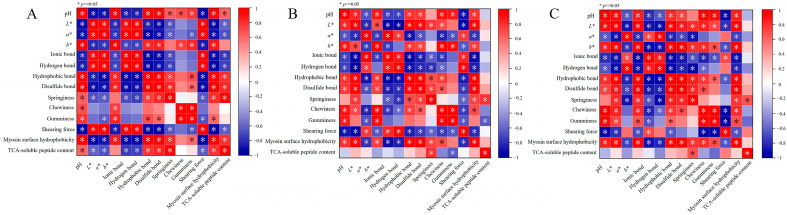
Pearson correlation analysis of muscle quality and protein function in (**A**) hooked hairtail (HH), (**B**) trawl-net hairtail (TH), and (**C**) radar-net hairtail (RH) during thermal processing.

The heatmap (Figure 6) depicted the correlation analysis between muscle quality and protein function, showcasing positive correlations in red and negative correlations in blue [52]. As shown in Figure 6, the surface hydrophobicity of the three different kinds of hairtail was significantly positively correlated with ionic bonds and hydrogen bonds and negatively correlated with hydrophobic bonds and disulfide bonds, suggesting that the spatial structure of the protein was closely related to the functional properties of the protein. Meanwhile, the a* of the three kinds of hairtail displayed a significant positive correlation with ionic bonds and hydrogen bonds and a negative correlation with hydrophobic bonds, disulfide bonds, and surface hydrophobicity. b* displayed a significant positive correlation with hydrophobic bonds, disulfide bonds, and surface hydrophobicity, and a significant negative correlation with ionic bonds. The results proved that the changes in the color of HH, TH, and RH muscles during heat treatment were related to the destruction of muscle protein structure and functional properties.

In addition, ionic bonds and hydrogen bonds in three kinds of hairtail displayed a significant positive correlation with springiness. Hydrophobic bonds displayed a significant positive correlation with springiness and a negative correlation with shearing force. The surface hydrophobicity was significantly correlated with springiness and shearing force. It could be further inferred that the relative content of chemical bonds caused by thermal denaturation of the protein changes, resulting in changes in the three-dimensional spatial conformation of the protein, impaired function, and deterioration of the muscle texture of the hairtail. This study revealed a close correlation between muscle quality and protein function in three kinds of hairtail.

## 4. Conclusions

The muscle quality of HH, TH, and RH heated at 30, 50, 70, and 90 °C was investigated. With increasing temperature, varying degrees of deterioration in muscle quality were observed in three kinds of hairtail, including an increase in pH, L*, a*, chewiness, and gumminess, disruption of chemical bonds, enhancement of surface hydrophobicity, and myofibrillar blurring and gap widening, as well as a decrease in b* and shearing force. The RH sample exhibited superior structural integrity and protein stability, characterized by minimal protein degradation and stable tissue structure even at high temperatures, reflected in the lowest content of TCA-soluble peptide and the highest content of chemical bonds. Additionally, the RH muscle displayed the highest integrity in collagen fibers and muscle fibers compared to HH and TH, evidenced by the microstructural examination results at 90 °C. The results of correlation analysis indicated that muscle quality was closely related to the functional properties of the protein. This investigation provides a theoretical basis and valuable insights for the change in muscle quality in different types of hairtail during thermal processing. However, due to the limitations of time and experimental conditions, the difference and mechanism of flavor quality alteration in HH, TH, and RH during water bath heating were not explored in depth.

## Data Availability

The original contributions presented in the study are included in the article, further inquiries can be directed to the corresponding author.
